# Prospective Evaluation of Intensity of Symptoms, Therapeutic Procedures and Treatment in Palliative Care Patients in Nursing Homes

**DOI:** 10.3390/jcm9030750

**Published:** 2020-03-10

**Authors:** Daniel Puente-Fernández, Concepción B. Roldán-López, Concepción P. Campos-Calderón, Cesar Hueso-Montoro, María P. García-Caro, Rafael Montoya-Juarez

**Affiliations:** 1 Doctoral Program of Clinical Medicine and Public Health, University of Granada, 18071 Granada, Spain; danielpuentefdz@correo.ugr.es; 2 Department of Statistics and Operational Research, Faculty of Medicine, University of Granada, 1016 Granada, Spain; iroldan@ugr.es; 3 Alicante Biomedical Research Institute (ISABIAL), 03010 Alicante, Spain; 4 Department of Nursing, Faculty of Health Sciences, Mind, Brain and Behaviour Research Institute, University of Granada, 18016 Granada, Spain; cesarhueso@ugr.es (C.H.-M.); mpazgc@ugr.es (M.P.G.-C.); rmontoya@ugr.es (R.M.-J.)

**Keywords:** palliative care, nursing homes, symptom assessment, drug therapy, therapeutics, longitudinal studies

## Abstract

The aim of the study is to evaluate the intensity of symptoms, and any treatment and therapeutic procedures received by advanced chronic patients in nursing homes. A multi-centre prospective study was conducted in six nursing homes for five months. A nurse trainer selected palliative care patients from whom the sample was randomly selected for inclusion. The Edmonton Symptoms Assessment Scale, therapeutic procedures, and treatment were evaluated. Parametric and non-parametric tests were used to evaluate month-to-month differences and differences between those who died and those who did not. A total of 107 residents were evaluated. At the end of the follow-up, 39 had (34.6%) died. All symptoms (*p* < 0.050) increased in intensity in the last week of life. Symptoms were more intense in those who had died at follow-up (*p* < 0.05). The use of aerosol sprays (*p* = 0.008), oxygen therapy (*p* < 0.001), opioids (*p* < 0.001), antibiotics (*p* = 0.004), and bronchodilators (*p* = 0.003) increased in the last week of life. Peripheral venous catheters (*p* = 0.022), corticoids (*p* = 0.007), antiemetics (*p* < 0.001), and antidepressants (*p* < 0.05) were used more in the patients who died. In conclusion, the use of therapeutic procedures (such as urinary catheters, peripheral venous catheter placement, and enteral feeding) and drugs (such as antibiotics, anxiolytics, and new antidepressant prescriptions) should be carefully considered in this clinical setting.

## 1. Introduction

The World Health Organization (WHO) [1] and the European Association of Palliative Care (EAPC) [2] encourage an increase in the quality of dying in long-term care settings. In fact, several articles call for more research on end-of-life interventions in these centres, in order to improve care practice [3,4]. Meanwhile, nursing homes have become a plausible alternative in situations where the home is not the most suitable place for the end of life, due to clinical complexity or lack of resources [5].

Recent studies have indicated that there is a high prevalence of physical and psychological symptoms in nursing homes [3,6,7,8]. All of these symptoms increase in intensity and prevalence as the end of life approaches [4,9]. Most of the studies that have evaluated end-of-life symptoms in nursing homes are retrospective studies [3,6,9,10,11]. They may exhibit selection bias and problems caused by poorly recorded or unrecorded data. Prospective studies may be very helpful to properly assess the changes in symptom control when is death is about to occur.

Hospices in Spain are not widely developed, so end-of-life care must be provided by other institutions. In the case of elderly patients, this care is mostly provided by nursing homes. In these centres, most of the beds are privately funded (71%) [12], although some are partially government-funded. In Andalusia, only nursing homes with more than 60 beds are required to offer twenty-four hour nursing services and their own medical care [13].

Beyond this, little is known regarding routine therapeutic procedures and pharmacological treatments in palliative patients in nursing homes. In a recent retrospective study in Spanish hospitals, patients who were at the end stage of their lives received similar therapeutic and diagnosis procedures to acute care patients [14]. This is congruent with other papers published previously: procedures such as catheter insertion, the use of aspirators, and other actions that are common for patient care in a general hospital can make the difference between comfort and discomfort for end-of-life patients [8,15,16].

Regarding pharmacological treatments, a recent review highlighted that many patients continue to receive medications that are not prescribed as palliative treatments or for symptom control, despite being in the end stage of life [17]. A previous review [18] pointed out that few studies focus on pharmacological de-prescription in end-of-life and concluded that life expectancy is not often used as a criterion for medication discontinuation, even though unnecessary drugs might cause side effects that may increase suffering for patients.

In this context, the European Association of Palliative Care [19] emphasises that, in Spain, there are no specific documents on palliative care in long-term care facilities, nor publications regarding the provision of palliative care in this type of centres in Spain.

The purpose of this study is to prospectively explore perception of symptom control, pharmacological treatments, and therapeutic procedures received by palliative patients admitted to nursing homes in the last six months of life. This is one of the first studies to use a prospective approach, and the first one to show the end-of-life situation in Spanish nursing homes with this methodology. We hypothesize that, when death is near, intensity of symptoms and pharmacological treatments linked to symptom control will increase, whereas the frequency of routine therapeutic procedures will be the same as in previous months.

## 2. Experimental Section

### 2.1. Design

This is a multi-centre prospective study which has been conducted in nursing homes in Spain.

### 2.2. Sample

Six nursing homes were selected for convenience based on their institutional characteristics: Presence of a multidisciplinary team, the possible involvement of professionals, and the presence of both public and private beds. All centres included in the study have more than 50 beds. In each centre, one or two nurses with close knowledge of the patient who have been working at the nursing home for at least 6 months were responsible for data collection. All of the nurses that participated signed an informed consent form and received training prior to data collection. In order to control bias and to produce reliable data for the research, these professionals completed a training course designed to explain the study, to ensure that the same data collection methods were followed, and to avoid the dropout of patients at the follow-up stage. The research team was in contact with them via email, and they visited the centres regularly, i.e., at least once a month.

### 2.3. Recruitment

Each nursing home nurse recruited residents with chronic diseases that met the following criteria according to the Spanish Society of Palliative Care (SECPAL):

- Advanced, progressive, and incurable disease
- Little to no possibility of response to any specific treatment
- Presence of numerous problems or intense, multiple, multifactorial and changing symptoms
- Great emotional impact on patient, family, and staff
- Life expectancy limited to 6 months.

Within each nursing home, twenty patients were randomly selected among all the patients that met the aforementioned inclusion criteria. They were observed and the data of interest were recorded without interfering with the natural course of events. Data were collected between June 2016 and January 2017. All participants, patients, or representatives of patients (in the case of cognitively impaired patients) were fully informed and signed informed consent forms.

### 2.4. Data Collection Procedure

Nurses collected demographic and clinical information from the clinical records of the patients. A structured questionnaire was used to collect socio-demographic (age, gender, years in the centre, marital status, and number of children) and clinical (medical diagnosis, Charlson Comorbidity Index) data from patient records.

For the systematic symptom assessment, we used the Edmonton Symptom Assessment System (ESAS) [20]. The ESAS has been validated for both patient and care partner report in different settings, including those with older people with multiple morbidities [21]. ESAS was used regularly in all the nursing homes that participated in the study for symptom assessment. The patient version of the ESAS was self-administered by cognitively intact patients. For cognitively impaired residents, the professional version of the ESAS was completed by trained nurses. The relatives of the patients were not involved in data collection. Cognitive impairment was defined as the patient making three or more mistakes in the Pfeiffer test. The Pfeiffer test was used in all the nursing homes that participated in this study.

The prescription of therapeutic procedures such as urinary catheterisation, enteral feeding, peripheral venous catheter placement, use of aerosol sprays, oxygen therapy, and pharmacological treatments such as non-opioid analgesics, opioid analgesics, antibiotics, bronchodilators, corticosteroids, antiemetics, antihistamines, antidepressants, anxiolytics, hypnotics, and barbiturates was also evaluated.

Data were collected between June 2016 and January 2017. For this study, outcome data were collected from clinical records of the first month (T1) and of the following months (T2, T3, T4, and T5) if residents were still alive. For all the residents who died within these six months, the same data were collected from the clinical records of the last week before death (CT = closure test). All participants, residents, and the care partner were fully informed and signed informed consent forms.

### 2.5. Statistical Analysis

A descriptive analysis was carried out to describe the main characteristics of the study sample. Numerical variables were described with the mean and standard deviation (SD) and the median and interquartile range (P25-P75). Categorical variables were described using absolute frequencies and percentages. Quantitative data were assessed for normality using the Kolmogorov-Smirnov test, and all of the quantitative data collected were found to deviate significantly from the normal distribution (*p* < 0.001). Due to this, non-parametric inferential tests were used. Pearson’s chi-squared test was used to evaluate between-group differences and McNemar’s test was used to compare the prevalence rates. Wilcoxon’s signed-rank test was used in order to compare month-by-month the symptoms reported using the ESAS for nursing home residents. Statistical analyses were conducted using IBM SPSS v.24. p-values of less than 0.05 were considered to be statistically significant.

### 2.6. Statement of Ethics

All participants (or when appropriate, a representative) signed a form to give their informed consent. The study received the approval of the Research Ethics Committee (PI-0619-2011). In compliance with Spanish Law (Article 16, Law 41/2002), patients’ data were anonymised.

## 3. Results

Thirteen patients dropped out of the study. Two of them moved to another nursing home. Ten patients or representatives of patients refused to give their informed consent during follow-up. One of the residents died before the beginning of the follow-up. As a result, the final sample consisted of 107 residents. Most of them were women (63.6%) and they had a mean age of 84.6 (SD = 7.4) years. The characteristics can be seen in Table 1.

Residents who died within the follow-up period (n = 39, 34.6%) were generally older and widowers, had a higher Charlson comorbidity index (CCI), and had more peripheral vascular and thromboembolic diseases, stroke or other cerebral lesions, arterial hypertension, and arrhythmia.

### 3.1. Perception of Symptom Intensity

In the comparison from T1 to T5, the perception of intensity of all symptoms was scored as moderate, except nausea and dyspnoea, which were scored as mild. No statistical differences were found in symptom intensity between T1 and T2 to T5 (Table 2). However, all differences were found to be statistically significant between T1 and symptoms in the last week of life (CT). In the comparison with CT, the median ratings for nausea (*p* = 0.040) and depression (*p* = 0.033) increased by up to 2 points; pain (*p* = 0.026), fatigue (*p* = 0.003), drowsiness (*p* ≤ 0.001), dyspnoea (*p* ≤ 0.001), and insomnia (*p* = 0.032) increased by up to 3 points; anxiety (*p* = 0.001), poor appetite (*p* ≤ 0.001), and malaise (*p* = 0.004) increased in intensity by up to 4 points. In this case, all symptoms were scored as moderate except nausea, which was scored as mild, and fatigue and malaise, which were scored as severe.

Residents who died during the follow-up period rated symptom intensity as higher for all symptoms, compared to those who were alive for the entire duration (Table 3).

### 3.2. Therapeutic Procedures and Pharmacological Treatments

No statistical differences were found in the comparison of therapeutic procedures between T1 and T2 to T5. Nevertheless, the analysis showed significant differences between T1 and CT (Table 4). The most repeated procedures (oxygen therapy (*p* ≤ 0.001), use of aerosol sprays (*p* = 0.008), and peripheral venous catheter placement (*p* = 0.039)) had an increase of between 20 and 40 percentage points. Despite this, the percentage of procedures related to urinary catheters (*p* = 1000) and enteral feeding (*p* = 0.221) was not significantly different between T1 and CT. 

Regarding pharmacological treatments, no significant differences were found between T1 and T2 to T5. However, some statistical differences were found between T1 and CT (Table 4). Opioid analgesics (*p* ≤ 0.001), antibiotics (*p* = 0.004), bronchodilators (*p* = 0.003) had a significant increase in usage, that increase being of 45, 35, and 29 percentage points, respectively.

CT: Closure Test. Week before death. Statistical differences were found in the use of peripheral venous catheters (*p* = 0.022), aerosol sprays (*p* = 0.001), and oxygen therapy (*p* = 0.001) between the patients who died in the follow-up and those who survived (Table 5).

Finally, the comparison of pharmacological treatments showed differences for use of antibiotics (*p* < 0.001), bronchodilators (*p* < 0.0001), opioids (*p* < 0.001), corticosteroids (*p* = 0.007), antiemetics (*p* < 0.001), and antidepressants (*p* = 0.026) between those who died and the survivors (Table 5). The administration of these treatments was significantly greater in all deceased patients than in those who survived, except for antidepressants, whose usage was significantly lower.

## 4. Discussion

This is one of the first studies that prospectively describes the last months of life of nursing home residents, and the first that has been conducted in Spain. Our results suggest that there is a sudden increase in symptoms, therapeutic procedures, and pharmacological treatments in the last week of life, in comparison with previous follow-up times. In addition, an increasing number of invasive therapeutic procedures, which may result in decreased comfort for residents, was observed, such as urinary catheter placement, peripheral venous catheter placement, and enteral feeding. Similarly, increased drug use, such as antibiotics, anxiolytics, and new antidepressant prescriptions was also observed.

The perception of the intensity of symptoms remains stable between T1 and the following months, but increases substantially between T1 and the last week of life. This finding is consistent with the previous literature, which details a worsening of symptoms in the last days of life [6,9]. Nevertheless, it is necessary to point out that the consulted studies used prevalence, not intensity, to assess symptoms. Thompson et al. [10] conducted a prospective study in which they assessed pain in the last six months of life of residents in nursing homes, showing that the intensity of their pain remained stable during a short follow-up period, except in the last days of life, when it increased.

In the same way, in relation to therapeutic procedures, there are significant differences in the use of peripheral venous catheters, oxygen therapy, enteral feeding, and aerosol sprays in the last week of life compared to at T1. These differences are greater if we compare the therapeutic procedures between patients who died within the follow-up period and survivors. Regarding oxygen therapy and the use of aerosol sprays, Hendriks et al. [4] highlighted that, unlike what the results of the present study show, there was a decrease in the use of these procedures when death was near. Similarly, a retrospective study conducted in four Spanish hospitals [14] showed that oxygen therapy was a very frequently used intervention at the end of life. This study also reported that there is an increase in the use of peripheral venous catheters during the last days of life [14].

Enteral feeding is another intervention that might be considered to be futile [22], as this does not improve the wellbeing of patients in a significant way and may even be prolonging the dying process [22]. One of the factors that may influence the continuation of enteral feeding is that some professionals and relatives consider this intervention to be a measure of comfort that should not be removed [23].

With respect to urinary catheters, previous studies are not clear about the use of these interventions at the end of life. A literature review by Farrington et al. [24], which included clinical practice guidelines, pointed out that, even though some of the studies reviewed stated that urinary catheterisation could be used to provide comfort to patients, this procedure may cause or increase patient discomfort [24].

This could be interpreted as the performance of futile interventions in the last week of life in the nursing homes analysed.

As expected, the use of some medications linked to symptom control such as opioids, corticosteroids, and antiemetics increased in the last days of life. Opioids were one of the most used drugs in this study, which corresponds to what is described in the literature [4,9]. In relation to the use of non-opioid analgesics, Jansen et al., [25] unlike our study, reported an increase in the use of this group of drugs at the end of life.

On the other hand, there is a decrease in the use of antidepressants in the last week of life, although the consumption of other psychotropic drugs remains stable, compared to in previous months. The use of this kind of drug in end-of-life care is controversial: Some of them could be considered futile since they are not used to improve symptoms typical of the end of life [26]. The time delay before certain antidepressants have a noticeable effect is long (usually 4–6 weeks), so their usage may be considered futile for this reason. In fact, although psychotropic drugs may be indicated for the control of psycho-emotional symptoms, authors point out that they can cause undesirable side effects in the geriatric population and an increased risk of mortality [27].

Regarding the use of antibiotics at the end of life, our results indicate an increase in the last week of life. This may be due to the high percentage of patients with dementia in the sample, in whom infections are a common cause of death. Although, previous studies indicate that the use of antibiotics improves the prognosis of patients and the relief of symptoms [28,29,30], other studies provide evidence that not administering antibiotics improves comfort [31]. Furthermore, using antibiotics is not without risk in fragile patients with chronic diseases, due to drug reactions, drug-drug interactions, and infections [32]. Even so, there is no consensus as to whether or not they should be used at the end of life.

Furthermore, there has been no decrease in the prescription of drugs for any of the drugs evaluated. According to the consulted bibliography, one of those that would be expected to decrease according to current recommendations would be anxiolytics [33]. In our sample, the prescription of anxiolytics did not diminish at the end of life. According to Westbury [33], ‘these psychotropic agents should be prescribed cautiously, at the lowest therapeutic doses for as short a time as possible, and be monitored regularly’. The literature consulted shows that identification of the terminal state increases the likelihood of a de-prescription occurring [34]. In the case of nursing homes, this identification is critical for facilitating patients’ access to palliative care and, consequently, for improving the quality of care they receive, their satisfaction with it, and their symptoms [35]. Our results may be due to the lack of use of predictive survival tools that could be used in these centres. Therefore, in the absence of a prediction of the end of life, professionals do not question the utility of the interventions that can be carried out.

The present article tried to demonstrate part of the reality of the care provided by Spanish nursing homes, the study of which has had its importance emphasised by institutions, such as the EAPC. It would have been interesting to have assessed patient comfort, in order to clarify the suitability of controversial interventions, due to their possible futility in an end-of-life context. This work is a first approach to the end of life in Spanish nursing homes, being the stepping stone on which it can be developed into an intervention programme to improve end-of-life care in these centres. At the same time, it could well help to validate specific tools, in order to assess the quality of the dying process and to improve the detection of palliative care needs.

It should also be highlighted that some limitations of this study may affect the reliability of our results. It should be noted that the sample size is small in comparison with other published studies, so it has not been possible to complete further analyses. Furthermore, characteristics of this study’s sample are similar to those in other studies conducted in nursing homes regarding age, sex, and diseases [4,6,9,36], so the results should be extrapolated carefully.

In this study, SECPAL criteria were used for case selection. Our results pointed out that only the 36.4% of patients of the sample have died, so a discussion on whether these criteria are the most appropriate is needed, particularly the limitation of a life expectancy of six months.

Several tools have been proposed to identify palliative care needs and prognosis [37]. For instance, White et al. [38] highlighted in a meta-analysis that the accuracy of the ‘Surprise Question’ referring to a one-year period was higher than 70% in trained professionals. For further studies, a year-long follow-up period could be considered.

## 5. Conclusions

In this prospective study, intensity of end-of-life symptoms increased in the last week of life. There is also an increase in therapeutic procedures and pharmacological treatments, but not all the procedures and drugs are linked to symptom management. Interventions (such as urinary catheters, peripheral venous catheter placement, and enteral feeding) and drugs (such as antibiotics, anxiolytics, and new antidepressant prescriptions) should be carefully considered in this clinical setting, in order to improve patient comfort and avoid futile treatments.

Primary care workers and stakeholders might support nursing home professionals in order to provide better symptom control and decide which interventions and drugs are to be recommended in the last days of life.

## Figures and Tables

**Table 1:** Socio-demographic and clinical characteristics of the patients.

Socio-Demographic and Clinical Characteristics of the Patients.	Total Sample *n* = 107		Dying within 6 month *n* = 39		Alive ≥ 6 month *n* = 68		*p*
Age, md (P25-P75)	84	(81–89)	86	(83–95)	84	(78.5–89)	0.011^1^
Female, *n* (%)	68	(63.6)	24	(64.8)	44	(62.9)	0.835^1^
Years in the centre, md (P25-P75)	2	(1–4)	2	(0.6–5)	2	(1.3–4)	0.946^2^
Marital status widower, *n* (%)	63	(60.8)	25	(67.6)	38	(54.3)	0.012^2^
Number of children, md (P25-P75)	2	(0–3)	2	(0–2)	3	(2–5)	0.222^1^
CCI, md (P25-P75)	3.5	(2–6)	4	(4–6)	3	(2–5)	0.007^1^
Primary diagnosis							
Myocardial infarction, *n* (%)	6	(5.6)	2	(5.3)	4	(6.0)	1.000^2^
Heart failure, *n* (%)	28	(26.2)	12	(31.6)	16	(23.3)	0.492^2^
Peripheral vascular disease, *n* (%)	10	(9.3)	9	(23.7)	1	(1.5)	0.000^2^
Thromboembolic disease, *n* (%)	7	(6.5)	6	(15.8)	1	(1.5)	0.009^2^
Stroke or other cerebral lesions, *n* (%)	45	(42.1)	22	(57.9)	23	(34.3)	0.024^2^
Hemiplegia, *n* (%)	14	(13.1)	7	(18.4)	7	(10.4)	0.370^2^
Arterial hypertension, *n* (%)	63	(58.9)	15	(38.5)	41	(60.3)	0.044^2^
Dementia	51	(47.7)	20	(52.6)	43	(64.2)	0.301^2^
COPD, *n* (%)	25	(23.4)	12	(31.6)	13	(19.4)	0.233^2^
Arrhythmia, *n* (%)	21	(19.6)	14	(36.8)	7	(10.4)	0.002^2^
Renal disease, *n* (%)	19	(17.8)	6	(15.8)	13	(19.4)	0.794^2^
Diabetes, *n* (%)	34	(31.8)	10	(27.8)	24	(38.1)	0.380^2^
Tumour, *n* (%)	17	(15.9)	8	(20.5)	9	(13.2)	0.308^2^
Solid tumour with metastasis, *n* (%)	10	(9.3)	4	(10.5)	6	(9.0)	1.000^2^

Charlson Comorbidity Index, CCI; ^1^ Mann-Whitney *U*-test; ^2^ Pearson’s chi-squared; COPD: chronic obstructive pulmonary disease.

**Table 2:** Month-by-month comparison of symptoms using Edmonton Symptom Assessment System (ESAS) for residents in nursing homes.

Symptoms	T1 vs. T2 (*n* = 102)		T1 vs. T3 (*n* = 95)		T1 vs. T4 (*n* = 84)		T1 vs. T5 (*n* = 82)		T1 vs. CT (*n* = 39)	
	md (P25-P75)	*p* ^1^	md (P25-P75)	*p* ^1^	md (P25-P75)	*p* ^1^	md (P25-P75)	*p* ^1^	md (P25-P75)	*p* ^1^
Pain										
T1	4 (2–6)	0.563	4 (2–6)	0.934	5 (2–7)	0.718	4 (2–6.5)	0.741	4 (2–7)	0.026
T(2-5) or CT	3.5 (2–6)		3.5 (2–6)		5 (2–7)		5 (2–7.5)		7 (2–9.5)	
Fatigue										
T1	5.5 (3–7)	0.225	5 (2–7)	0.485	5.5 (3–7)	0.443	4 (2.5–8)	0.559	5 (3–7)	0.003
T(2-5) or CT	5.5 (3–8)		5 (2–8)		5.5 (3–8)		5 (2.5–9)		8 (3.5–9)	
Nausea										
T1	0 (0–3)	0.721	0 (0–2)	0.728	0 (0–2.5)	0.228	0 (0–2)	0.836	0 (0-3)	0.040
T(2-5) or CT	0 (0–3)		0 (0-2)		0 (0–1)		1 (0–3)		2 (0–7)	
Depression										
T1	3 (0–6)	0.773	3 (0–6)	0.833	3 (0–6)	0.654	3 (0–6)	0.589	3 (0–7)	0.033
T(2-5) or CT	3 (0–5.5)		2 (0–7)		3.5 (1–6)		3.5 (1–7)		4.5 (1–9)	
Anxiety										
T1	3 (0–6)	0.298	3 (0–5.5)	0.470	3 (0–6)	0.889	3 (0–6)	0.553	3 (0–6)	0.001
T(2-5) or CT	2 (0–5)		2 (0–6)		3 (0–6)		4 (0–6)		7 (1–9)	
Drowsiness										
T1	2 (4–7)	0.357	4 (2–7)	0.777	3.5 (2–7)	0.985	3 (0.5–5)	0.850	4 (3–6)	<0.001
T(2-5) or CT	2 (1–7)		4 (1–6)		4 (1–7)		4 (1–5)		7 (6–10)	
Poor appetite										
T1	3 (0–6)	0.624	3 (0–6)	0.479	3 (0–7)	0.332	3 (0–6)	0.473	3 (1–7)	<0.001
T(2-5) or CT	4 (0–6)		2 (0–5.5)		2 (0–6.5)		2 (0–4)		7 (3–10)	
Malaise										
T1	5 (0–7)	0.114	5 (0–7)	0.284	5 (0–7)	0.210	4 (0–7)	0.357	5 (0–7)	0.004
T(2-5) or CT	4.5 (0–6)		4 (0–6)		5 (1–8)		4 (3–7)		9 (2–9.5)	
Dyspnoea										
T1	1 (0–6)	0.522	1 (0–6)	0.765	1 (0–6)	0.602	1 (0–6)	0.187	4 (0–6)	<0.001
T(2-5) or CT	1 (0–5)		1 (0–6)		0 (0–6.5)		0 (0–5.5)		7 (5–9)	
Insomnia										
T1	2.5 (0–6)	0.991	2 (0–6)	0.480	2.5 (0–7)	0.119	2 (0–6)	0.955	3 (0–7)	0.032
T(2-5) or CT	2 (0–6)		2 (0–5)		3 (0–6)		3 (0–6)		6 (1-9)	

Wilcoxon’s signed-rank test^1^; T1: Initial follow-up time; T2, T3, T4, T5: Different follow-up times; CT: Closure Test. Week before death; P25: 25th percentile; P75: 75th percentile.

**Table 3:** Comparison of symptoms using ESAS in residents of nursing homes who died with those who did not die.

Symptoms	Dying within 6 months *n* = 39 n(Range)	Alive ≥ 6 months *n* = 68 n(Range)	*p* ^1^
Pain, md (P25-P75)	7 (2–9)	4 (2–6)	0.012
Fatigue, md (P25-P75)	8 (3.5–9)	6 (3–8)	0.005
Nauseas, md (P25-P75)	1 (0–7)	0 (0–1)	0.003
Depression, md (P25-P75)	4 (1–9)	3 (0–6)	0.050
Anxiety, md (P25-P75)	4 (1–9)	3 (0–6)	0.002
Drowsiness, md (P25-P75)	7 (1–9)	4 (0–7)	< 0.001
Poor appetite, md (P25-P75)	7 (6–10)	4 (2–7)	< 0.001
Malaise, md (P25-P75)	9 (2–9.5)	5 (2–7)	< 0.001
Dyspnoea, md (P25-P75)	7 (5–9)	1 (0–6)	< 0.001
Insomnia, md (P25-P75)	6 (1–9)	2 (0–6)	0.011

^1^Mann-Whitney *U*-test.

**Table 4:** Comparison of therapeutic procedures and pharmacological treatments by months for patients in nursing homes.

Therapeutic Procedures/Pharmacological Treatments	T1 vs. T2 (*n* = 102)		T1 vs. T3 (*n* = 95)		T1 vs. T4 (*n* = 84)		T1 vs. T5 (*n* = 82)		T1 vs. CT (*n* = 37)		
	%	*p* ^1^	%	*p* ^1^	%	*p* ^1^	%	*p* ^1^	%	*p* ^1^	95% CI^2^
Therapeutic procedures											
Urinary catheter											
T1	14.7	1.000	14.7	1.000	11.9	0.752	14.6	0.267	21.1	1.000	
T(2-5) or CT	13.7		14.7		14.3		8.5		23.7		
Peripheral venous catheter placement											
T1	26.5	0.860	24.2	0.522	22.9	0.502	24.4	0.480	25.6	0.039	4.1–39.9
T(2-5) or CT	24.5		28.4		18.6		19.5		48.7		
Enteral feeding											
T1	11.8	1.000	14.0	1.000	15.5	1.000	14.6	0.789	5.3	0.221	
T(2-5) or CT	11.8		14.0		15.5		17.1		15.8		
Aerosol sprays											
T1	23.5	0.789	18.9	0.248	19.3	0.267	18.3	0.248	28.2	0.008	11.5–51.9
T(2-5) or CT	21.6		26.4		25.3		22.0		61.5		
Oxygen therapy											
T1	30.4	0.803	28.4	0.450	27.4	0.511	24.4	0.343	36.9	<0.001	17.6–65.3
T(2-5) or CT	32.4		31.9		33.3		29.3		79.5		
Pharmacological treatments											
Non-opioid analgesics											
T1	58.8	0.263	54.7	0.345	54.8	0.404	51.2	0.170	71.8	0.628	
T(2-5) or CT	64.7		61.1		60.7		61.0		64.1		
Opioid analgesics											
T1	12.7	0.375	11.6	0.131	8.3	0.445	12.2	1.000	17.9	<0.001	25.6-57.3
T(2-5) or CT	15.7		16.8		11.9		11.0		61.5		
Antibiotics											
T1	21.6	0.185	20.0	0.109	21.4	0.136	17.1	0.211	30.8	0.004	14.9–53.4
T(2-5) or CT	29.4		29.5		31.0		25.6		66.7		
Bronchodilators											
T1	27.5	0.302	26.3	0.114	28.6	0.814	32.9	0.505	25.6	0.003	12.4–41.2
T(2-5) or CT	32.4		32.6		31.0		29.3		53.8		
Corticosteroids											
T1	20.6	1.000	18.9	1.000	15.5	0.453	17.1	1.000	28.2	0.267	
T(2-5) or CT	21.6		20.0		20.2		18.3		41.0		
Antiemetics											
T1	7.8	1.000	9.5	1.000	6.0	1.000	8.5	0.505	17.9	0.227	
T(2-5) or CT	7.8		9.5		7.1		12.2		30.8		
Antihistamines											
T1	7.8	1.000	6.3	0.500	6.0	1.000	3.7	1.000	10.3	0.248	
T(2-5) or CT	7.8		8.4		7.1		3.7		2.6		
Antidepressants											
T1	33.3	1.000	34.7	0.617	32.5	0.479	33.7	0.131	28.2	0.114	
T(2-5) or CT	33.3		32.6		30.1		27.7		12.8		
Anxiolytics											
T1	32.4	0.773	35.0	0.181	28.6	0.752	29.3	0.386	41.0	0.150	
T(2-5) or CT	30.4		28.4		25.7		34.1		25.6		
Hypnotics/barbiturates											
T1	49.0	0.267	46.3	1.000	41.7	0.383	50.0	0.424	51.3	1.000	
T(2-5) or CT	44.1		46.3		47.6		43.9		71.8		

^1^McNemar’s test; ^2^Agresti Min 95% confidence interval for p2-p1.; T1: Initial follow-up time.; T2, T3, T4, T5: Different follow-up times.

**Table 5:** Comparison of therapeutic procedures and pharmacological treatments in nursing home patients who died with those who did not die.

Therapeutic Procedures/Pharmacological Treatments	Dying within 6 months, *n* = 39	Alive ≥ 6 months, *n* = 68	p*	OR (95% CI)
Therapeutic procedures				
Urinary catheter, (%)	23.7	13.2	0.176	
Peripheral venous catheter placement, (%)	48.7	25.0	0.022	2.850 (1.238–6.562)
Enteral feeding, (%)	15.8	14.7	0.867	
Aerosol sprays, (%)	61.5	20.6	<0.001	6.171 (2.578–14.771)
Oxygen therapy, (%)	79.5	26.5	<0.001	10.764 (4.181–27.713)
Pharmacological treatments				
Non-opioid analgesics	65.8	54.4	0.350	
Opioid analgesics	63.2	10.3	<0.001	14.939 (5.372–41.546)
Antibiotics	65.8	16.2	<0.001	9.965 (3.930–25.268)
Bronchodilators	55.3	16.2	<0.001	6.401 (2.580–15.880)
Corticosteroids	42.1	16.2	0.007	3.769 (1.514–9.379)
Antiemetics	31.6	4.4	<0.001	10.000 (2.607–38.359)
Antihistamines	2.6	7.4	0.417	
Antidepressants	13.2	33.3	0.026	.278 (0.096–0.805)
Anxiolytics	26.3	29.4	0.909	
Hypnotics/barbiturates	52.6	48.5	0.839	

*Pearson’s chi-squared; OR (95% CI), odds ratio (95% confidence interval of the odds ratio).

## References

[1] Hall S., Petkova H., Tsouros A.D., Costantini M., Higginson I.J., World Health Organization (2011). Palliative Care for Older People: Better Practices.

[2] Froggatt K.A., Reitinger E., Heimerl K., Hockley J., Brazil K. (2013). Palliative Care in Long-Term Care Settings for Older People: EAPC Taskforce. Eur. J. Palliat. Care.

[3] Hendriks S.A., Smalbrugge M., Hertogh C.M.P.M., Van Der Steen J.T. (2014). Dying with dementia: Symptoms, treatment, and quality of life in the last week of life. J. Pain Symptom Manag..

[4] Hendriks S.A., Smalbrugge M., Galindo-Garre F., Hertogh C.M.P.M., van der Steen J.T. (2015). From Admission to Death: Prevalence and Course of Pain, Agitation, and Shortness of Breath, and Treatment of These Symptoms in Nursing Home Residents with Dementia. J. Am. Med. Dir. Assoc..

[5] Costa V., Earle C.C., Esplen M.J., Fowler R., Goldman R., Grossman D., Levin L., Manuel D.G., Sharkey S., Tanueputro P. (2016). The determinants of home and nursing home death: A systematic review and meta-analysis. BMC Palliat. Care.

[6] Estabrooks C.A., Hoben M., Poss J.W., Chamberlain S.A., Thompson G.N., Silvius J.L., Norton P.G. (2015). Dying in a Nursing Home: Treatable Symptom Burden and its Link to Modifiable Features of Work Context. J. Am. Med. Dir. Assoc..

[7] Sandvik R.K., Selbaek G., Bergh S., Aarsland D., Husebo B.S. (2016). Signs of Imminent Dying and Change in Symptom Intensity During Pharmacological Treatment in Dying Nursing Home Patients: A Prospective Trajectory Study. J. Am. Med. Dir. Assoc..

[8] Hoben M., Chamberlain S.A., Knopp-Sihota J.A., Poss J.W., Thompson G.N., Estabrooks C.A. (2016). Impact of Symptoms and Care Practices on Nursing Home Residents at the End of Life: A Rating by Front-line Care Providers. J. Am. Med. Dir. Assoc..

[9] Koppitz A., Bosshard G., Schuster D.H., Hediger H., Imhof L. (2015). Type and course of symptoms demonstrated in the terminal and dying phases by people with dementia in nursing homes. Zeitschrift für Gerontol und Geriatr..

[10] Thompson G.N., Doupe M., Reid R.C., Baumbusch J., Estabrooks C.A. (2017). Pain Trajectories of Nursing Home Residents Nearing Death. J. Am. Med. Dir. Assoc..

[11] Smedbäck J., Öhlén J., Årestedt K., Alvariza A., Fürst C.J., Håkanson C. (2017). Palliative care during the final week of life of older people in nursing homes: A register-based study. Palliat. Support Care.

[12] Campos-Calderón C., Montoya-Juárez R., Hueso-Montoro C., Hernández-López E., Ojeda-Virto F., García-Caro M.P. (2016). Interventions and decision-making at the end of life: The effect of establishing the terminal illness situation. BMC Palliat. Care.

[13] Centro de Investigaciones Sociológicas (2018). Informe Envejecimiento en Red. Centro de Investigaciones Sociológicas. Madrid. http://envejecimiento.csic.es/documentos/documentos/enred-estadisticasresidencias2017.pdf.

[14] (2007). Decreto 168/2007 de 12 de Junio, por el que se Regula el Procedimiento para el Reconocimiento de la Situación de Dependencia y del Derecho a las Prestaciones del Sistema para la Autonomía y Atención a la Dependencia, así como los órganos Competentes para su Valoración. Boletín Oficial de la Junta de Andalucía.

[15] Verhofstede R., Smets T., Cohen J., Eecloo K., Costantini M., Van Den Noortgate N., Deliens L. (2017). End-of-Life Care and Quality of Dying in 23 Acute Geriatric Hospital Wards in Flanders, Belgium. J. Pain Symptom Manag..

[16] Li Q., Zheng N.T., Temkin-Greener H. (2013). Quality of end-of-life care of long-term nursing home residents with and without dementia. J. Am. Geriatr. Soc..

[17] Poudel A., Yates P., Rowett D., Nissen L.M. (2017). Use of Preventive Medication in Patients with Limited Life Expectancy: A Systematic Review. J. Pain Symptom Manag..

[18] Tjia J., Velten S.J., Parsons C., Valluri S., Briesacher B.A. (2013). Studies to Reduce Unnecessary Medication Use in Frail Older Adults: A Systematic Review. Drugs Aging.

[19] Arias-Casais N., Garralda E., Rhee J., Lima L., Pons-Izquierdo J., Clark D., Hasselaar J., Ling J., Mosoiu D., Centeno-Cortes C. (2019). EAPC Atlas of Palliative Care in Europe.

[20] Carvajal A., Centeno C., Watson R., Bruera E. (2011). A comprehensive study of psychometric properties of the Edmonton Symptom Assessment System (ESAS) in Spanish advanced cancer patients. Eur. J. Cancer.

[21] Nekolaichuk C., Watanabe S., Beaumont C. (2008). The Edmonton Symptom Assessment System: A 15-year retrospective review of validation studies (1991–2006). Palliat. Med..

[22] Krishna L. (2011). Nasogastric feeding at the end of life: A virtue ethics approach. Nurs. Ethics..

[23] Van der Riet P., Good P., Higgins I., Sneesby L. (2008). Palliative care professionals’ perceptions of nutrition and hydration at the end of life. Int. J. Palliat. Nurs..

[24] Farrington N., Fader M., Richardson A. (2019). Managing urinary incontinence at the end of life: An examination of the evidence that informs practice. Int. J. Palliat. Nurs..

[25] Jansen K., Schaufel M.A., Ruths S. (2014). Drug treatment at the end of life: An epidemiologic study in nursing homes. Scand. J. Prim. Health Care.

[26] McNeil M.J., Kamal A.H., Kutner J.S., Ritchie C.S., Abernethy A.P. (2016). The Burden of Polypharmacy in Patients near the End of Life. J. Pain Symptom Manag..

[27] Park Y., Franklin J.M., Schneeweiss S., Levin R., Crystal S., Gerhard T., Huybrechts K.F. (2015). Antipsychotics and mortality: Adjusting for mortality risk scores to address confounding by terminal Illness. J. Am. Geriatr. Soc..

[28] Rosenberg J.H., Albrecht J.S., Fromme E.K., Noble B.N., McGregor J.C., Comer A.C., Furuno J.P. (2013). Antimicrobial use for symptom management in patients receiving hospice and palliative care: A systematic review. J. Palliat. Med..

[29] Lam P.T., Chan K.S., Tse C.Y., Leung M.W. (2005). Retrospective analysis of antibiotic use and survival in advanced cancer patients with infections. J. Pain Symptom Manag..

[30] Oh D.Y., Kim J.H., Kim D.W., Im S.A., Kim T.Y., Heo D.S., Kim N.K. (2006). Antibiotic use during the last days of life in cancer patients. Eur. J. Cancer Care (Engl.).

[31] Givens J.L., Jones R.N., Shaffer M.L., Kiely D.K., Mitchell S.L. (2010). Survival and comfort after treatment of pneumonia in advanced dementia. Arch. Intern Med..

[32] Juthani-Mehta M., Malani P.N., Mitchell S.L. (2015). Antimicrobials at the End of Life: An Opportunity to Improve Palliative Care and Infection Management. JAMA.

[33] Westbury J.L., Gee P., Ling T., Brown D.T., Franks K.H., Bindoff I., Peterson G.M. (2018). RedUSe: Reducing antipsychotic and benzodiazepine prescribing in residential aged care facilities. Med. J. Aust..

[34] Van Den Noortgate N.J., Verhofstede R., Cohen J., Piers R.D., Deliens L., Smets T. (2016). Prescription and Deprescription of Medication during the Last 48 Hours of Life: Multicenter Study in 23 Acute Geriatric Wards in Flanders, Belgium. J. Pain Symptom Manag..

[35] Stephens C.E., Hunt L.J., Bui N., Halifax E., Ritchie C.S., Lee S.J. (2018). Palliative Care Eligibility, Symptom Burden, and Quality-of-Life Ratings in Nursing Home Residents. JAMA Intern Med..

[36] Brandt H.E., Ooms M.E., Deliens L., van der Wal G., Ribbe M.W. (2006). The last two days of life of nursing home patients-a nationwide study on causes of death and burdensome symptoms in the Netherlands. Palliat. Med..

[37] Simmons C.P.L., McMillan D.C., McWilliams K., Sande T.A., Fearon K.C., Tuck S., Fallon M.T., Laird B.J. (2017). Prognostic Tools in Patients with Advanced Cancer: A Systematic Review. J. Pain Symptom Manag..

[38] White N., Kupeli N., Vickerstaff V., Stone P. (2017). How accurate is the ‘Surprise Question’ at identifying patients at the end of life? A systematic review and meta-analysis. BMC Med..

